# Social Prescribing—An Effort to Apply a Common Knowledge: Impelling Forces and Challenges

**DOI:** 10.3389/fpubh.2020.515469

**Published:** 2020-11-27

**Authors:** M. Mofizul Islam

**Affiliations:** Department of Public Health, La Trobe University, Melbourne, VIC, Australia

**Keywords:** social prescribing, non-medical care, referral to social services, social determinants of health, link worker, community referral

## Abstract

In recent times, social prescribing has been introduced in some countries, and substantially in the U.K. The objective of this scheme is to offer non-medical care mainly to primary care patients. Although the idea of this scheme is not new, its formalization is. Using a narrative synthesis of peer-reviewed and gray literature, this article discusses the social prescribing scheme, some of its compelling aspects and challenges in offering non-medical care, particularly regarding referrals being made from primary care settings. The social prescribing scheme has several impelling forces that include its potential to turn primary care to primary healthcare, tackle social determinants of health and social needs, improve wellbeing and physical health, offer person-centered care, strengthen preventive care, and bridge healthcare organizations with the third sector. This scheme also faces several challenges including service standards and boundaries, sustainability, availability of appropriate services, low engagement of clients and insufficient evidence. While this scheme lacks validated evidence, it is theoretically compelling. Given that the demand for non-medical care is growing in most societies and that the usefulness of non-medical care is gaining prominence, social prescribing is likely to continue to proliferate.

## Introduction

In many places, a substantial proportion of primary care patients consult general practitioners (GPs) for problems that are primarily non-medical (1, 2). Supporting people whose health problems are exacerbated by non-medical issues is a challenge for the healthcare system. However, the willingness and ability of healthcare professionals to take patients' daily lives and concerns into account are seen as key element of good quality medical care (3). Indeed, primary care patients may not be able to differentiate between medical and non-medical problems, since these are often intricately connected (4), while primary care workers may not be equipped to handle such problems (also termed as social problems) (5). As a result, patients may assess overall care as being inadequate and feel dissatisfied. Growing demand for holistic support, including non-medical care, has recently prompted the emergence of social prescribing initiatives in diverse national contexts.

Social prescribing is a generalized term that originated in the U.K. It is also known as social/community referral (6). The Social Prescribing Network defines social prescribing as “enabling healthcare professionals to refer patients to a link-worker, to co-design a nonclinical social prescription to improve their health and wellbeing” (7). The King's Fund (8) defines it as a mechanism for enabling primary care professionals to refer people to nonclinical services in their local areas. The CentreForum Mental Health Commission broadens the definition to include the mechanism of linking patients to social services regardless of the sources of the referrals (9). Therefore, social prescribing is a non-medical referral option for GPs, for other medical and some non-medical professionals and also for self-referral to the sources of support.

The referral mechanisms, target groups, services offered through social prescribing vary across settings (6, 10, 11). However, the process usually involves screening for non-medical needs and referrals to support services that are typically offered by community-based organizations. In the U.K., patients are generally referred to “link-workers,” who work with them and mediate between the referrer and the service provider. Services may include support and advice on physical activity, loneliness, social networking, job hunting, housing, financial hardship, debt, learning new skills, legal issues, opportunities to participate in arts and other creative activities, volunteering, mutual aid and parenting (6, 10, 11). Approaches to social prescribing vary from “small-scale” to “comprehensive” (11).

The literature on social prescribing is sparse and based mainly on operational elements. However, as a concept and model, social prescribing, despite its many challenges, has proliferated without a concomitant evidence base (12), primarily because of its theoretically compelling underpinnings. This article uses available literature to describe and discuss social prescribing, some of its compelling aspects and challenges in offering non-medical care, particularly regarding referrals from primary care settings.

## Social Prescribing in Primary Care

Social prescribing is an effort to apply the common knowledge that people's health is largely determined by socioeconomic factors, and that people who have access to social supports within their communities are healthier (13). These factors are beyond the service scope of healthcare professionals, but account for more than half of the determinants of health and wellbeing. The social prescribing effort is, in fact, the formalization of the process, as many healthcare professionals have already been undertaking similar activities although informally or somewhat on *ad hoc* basis. Although people can find and access non-medical services independently, a formal referral (i.e., officially sending or directing patients) brings importance to the referred-services and gives it the credibility afforded to health professionals. Formal referrals underline the “health value” of the service and legitimize the problems and their importance (14, 15). Furthermore, referrals help patients to be “transferred” from healthcare settings to appropriate services/resources (16). Also, without formal structures, patients may not use their referrals (5).

As described above, there is no agreed definition of social prescribing. Although an agreed definition is not essential for patients, it is for key stakeholders such as clients, clinicians, social service providers, link-workers, funders. Lack of clarity regarding the concept may negatively impact the development of relevant services. For instance, it is still not entirely clear who the prescriber is. Sometimes healthcare professionals and other times link-workers are identified as social prescribers (17–20). Ideally, healthcare professionals would make the referrals, with link-workers helping the clients to select the appropriate services. The community organizations are the service providers, not the prescribers. Thus, the term “social prescribing” is ambiguous; perhaps, “community referral” is better.

## Link-Worker in Social Prescribing

Healthcare professionals have limited time and capacity to help patients with their non-medical needs, so the provision of link-workers was introduced in the U.K.-based scheme. Also, factors such as short-term, precarious funding can lead to closures, mergers, arrivals and the renaming of services (21), rendering local directories out of date. Link-workers are expected to possess up-to-date information and connect the organizations working across a neighborhood. Furthermore, for socially isolated patients, only flagging or referral for social services may not be enough. Link-workers provide initial support, for instance; they may accompany the clients in their first visit, facilitate the navigation from healthcare to appropriate social services or work with patients to make plans (19) and with clinicians to generate referrals and provide updates on patients' progress (10).

## Social Prescribing in Non-U.K. Settings

This scheme is also attracting interest in Ireland (22), the United States (23), Canada (24), Australia (25–27), the Netherlands (28), and some parts of Scandinavia (29, 30). Many GPs and allied health professionals in Australia reported that they sometimes or often make referrals for non-health services in the community (31). Services are sometimes offered to specific groups of clients; for instance, a scheme in Australia tries to link injured workers with non-medical supports within their communities (32). In some settings, both support services and community referrals are offered from the same primary healthcare facilities, although this is not often described as social prescribing. For example, Australia, Canada, U.K. and USA have low-threshold primary healthcare services for homeless and people who use illicit drugs (33, 34). These facilities offer advice and referral for social and welfare services, internet/telephone facilities, rest-rooms, washrooms, snacks and coffee, and legal services (34).

Little literature exists on social prescribing within developing countries (35), most of which have limited community and voluntary sectors. However, in many developing countries, non-government and not-for-profit organizations operate and try to improve the socio-economic and health statuses of the vulnerable people (36). Some of these organizations may well be able to work closely with the primary healthcare sectors to offer social and welfare services.

## Some Impelling Forces of Social Prescribing Scheme

### Progressing From Primary Care to Primary Healthcare

A well-designed social prescribing scheme can upgrade primary care to “primary healthcare” and can change the family physician type care for individuals to a service provision committed to community health development (37). Primary care usually involves a single service, intermittent management of specific illnesses for an individual and follows a time-limited appointment (38). The Alma-Ata declaration states that the primary healthcare relies, at local and referral levels, on medical and non-medical services and auxiliaries and community workers as applicable (39). It also states that community participation is a key aspect of primary healthcare. This scheme can build community resilience, social capital, and a health-generating environment with solutions to suit local needs and aspirations and alleviate health problems (5).

### Tackle Social Determinants of Health and Social Needs

It is now well-recognized that people's health is determined not so much by what healthcare professionals do for patients, but by arrangements in society (40). The importance of tackling social determinants of health and health inequalities is paramount. A high prevalence of primary care consultations for non-medical problems suggests that something needs to be done and highlights the importance of the social model of health (41). Social prescribing can help patients tackle some of these social determinants with referrals to support services (42).

Health professionals may not see any benefits of screening social determinants of health unless they can do something to help patients tackle them (43). Access to social services for patients through this scheme can encourage healthcare providers to make the screening of social determinants of health a part of their care process (44). A business case for social support under healthcare investment is gradually evolving (45).

The Marmot Review outlined the importance of social prescribing (46). The aspirations of this scheme and what “social determinants of health” want to tackle are common (47). However, this scheme is not a magic bullet (48), the macro-economic policies and programmes to tackle health inequalities are beyond its scope.

### Improve Wellbeing and Physical Health

With an aging population, rising multimorbid chronic conditions and social isolation, and as the importance and demand for wellbeing grow (49) there is a strong theoretical and practical ground for social prescribing (50, 51). This scheme can facilitate social inclusion, physically and socially active life, behavioral change (e.g., smoking cessation, physical activity) and less reliance on medicine – all of which are pivotal for better health and wellbeing (46). Although the evidence for social prescribing on physical health is insufficient, it is relatively supportive of social and psychological wellbeing (52, 53). There is evidence that social prescribing reduces social isolation and anxiety; increases social engagement, confidence about health, life in general, and the capability to perform day-to-day activities (54, 55). Services through social prescribing can improve various components of “wellbeing” such as self-esteem, self-confidence, social interactions, day-to-day functioning, inclusion (52, 53, 56, 57), which, as the Whitehall Studies demonstrate, can then impact on physical health (51).

### More Toward Preventive Care in a Person-Centered Approach

Social prescribing is a preventive approach for patients to be more reliant on a healthy lifestyle, and less on medical care. It recognizes that if the social aspects of health can be tackled proactively, many illnesses could be prevented. Marmot (58) argues that the existing practice of giving advice only is unlikely to work at a population level. Social prescribing adds an option for GPs so that they do not need to be reliant only on medicines. Its advocates find this scheme fundamental to prevention (59). Indeed, early utilization of some services such as befriending may prevent loneliness and depression (28), and social networking and exercise can improve the quality of life, thereby helping in tertiary prevention (30).

Social prescribing is “organic” in its approach. Functioning alongside medical care, social prescribing provides an individualized approach, with patients supported to identify and achieve personalized goals (7) based on their strengths and resources available in their communities. This scheme recognizes that everyone has different needs – some people benefit from meeting new people, while others enjoy gaining new skills (56).

### Bridge Between Healthcare Organizations and Third Sector

The social prescribing scheme can facilitate linking health sector with the third sector [voluntary, community and social enterprise (VCSE) organizations]. Despite being a potential resource, voluntary sector support is known to be underused. The weak link between health services and VCSE organizations is a reason for that. Social prescribing gives patients access to social services in their communities. There exists a potential to nurture social capital in localities and catalyze it to make health-creating communities, wherein community members can take care of themselves and each other (60). By making social care needs tangible, social prescribing can empower patients to search for solutions to social difficulties that might affect their health (48, 60).

## Some Challenges ahead for Social Prescribing

The apparent simplicity of the concept of social prescribing can mask its challenges. This list of challenges is long (Table 1) and, understandably, varies across settings. Some challenges, likely to be generic in the current circumstances, are described below.

**Table 1:** Some impelling forces and challenges to the social prescribing scheme.

**Impelling forces**	**Challenges**
Progressing from primary care to primary healthcare	Setting standards and boundaries
Tackle social determinants of health and social needs	Sustainability
Improve wellbeing and physical health	Availability of appropriate services
More toward preventive care in a person-centered approach	Low engagement of clients
Bridge between healthcare organizations and third sector	Insufficient evidence in support of social prescribing

### Setting Standards and Boundaries

Currently, the social prescribing setup lacks clear guidance and reflects a “laissez-faire” attitude about service standards (48). There are no clear standards about the skillset of link-workers. Although a recent document discusses the common attributes of a good scheme and job description of link-workers, these attributes are yet to be evaluated (61). There are concerns regarding service standards and confidentiality in the third sector, in comparison with the mainstream health sector (15). Also, there is lack of clarity in service-boundaries (62). For instance, it is unclear as to who should manage the link-workers – healthcare professionals or the third sector (10). Similarly, whether patients' medical history should be transferred to community services, if deemed necessary, or left to patients' discretion is also unclear (63). An academic set-up with accreditation and continuing professional development may alleviate these unclarities and give professional recognition to those working in social prescribing (7).

Having professional standards is also essential for quality assurance. The facilities in the third sector vary substantially—such as self-help groups, charitable trusts and community interest companies—and so do their governance structures (19). The standards that exist in the voluntary sector may not be appropriate for social prescribing, and thus health professionals may not feel confident about the governance and professional standards of link-workers and facilities providing the services and safety of the patients (19). A clear line of accountability is needed at every stage of the social prescribing process and between the organizations and providers involved.

### Sustainability

Currently, the VCSE sector is the mainstay of this scheme. Thus, its success is arguably contingent upon the available local services or activities to which people can be referred (64) and upon the relationships between primary care and the VCSE sector. These relationships can be precarious and fragile (65). Several factors may potentially hinder sustainability. First, services are usually provided free-of-cost. Second, funding for these schemes is often non-recurrent, putting them at risk of ending abruptly. Funding reductions due to austerity measures and COVID-19 may further stretch the sustainability. Third, link-workers in many schemes are volunteers. There is a demand for a paid role with a pathway to career progression (66), which warrants additional funding. Fourth, to achieve a tangible benefit, clients need to be supported for an appropriate period (5, 16), which may increase further pressure on this sector. Indeed, some VCSE organizations already have significant commitments and may not have capacity to meet the additional demand created through social prescribing (67). Therefore, the sustainability is a looming question. However, the third sector is believed to have capacity to accommodate the demand of social prescribing, regardless of its volume (68). The validity of this claim needs to be evaluated.

### Availability of Appropriate Services

This scheme cannot work if the required services are unavailable. Often VCSEs are developed based on their own agenda and resources. Their service aims may not be aligned with the demand of clients referred through social prescribing. For instance, in some settings, financial matters (e.g., debt, general money-related struggles and managing money for food purchases) and advice on welfare benefits and housing are the most common reasons for referral (69), although these are not within the service scope of the schemes, and that the staff are not qualified to provide advice on these. Inadequate service has been identified as a barrier (68, 70). Given that some services need specific skillsets and substantial resources, it is not unlikely that social prescribing will gradually evolve out to offer only a certain type of service.

### Insufficient Evidence in Support of Social Prescribing

Robust evidence on the effectiveness of this scheme is limited (20, 62), which is attributable to several factors, including difficulties in evaluation (6); a relatively long time-lag for benefits to emerge; and costs required for evaluation (12). Usually, these schemes are “emerged” rather than being systematically designed with an innate evaluation plan at the outset (62), so not all schemes are evaluable (71). However, the lack of robust evidence does not mean social prescribing is ineffective. Given the complexities involved in the evaluation, it might be appropriate to evaluate individual components in various service models so that the findings can inform as to when these schemes are effective, by whom, for whom, and at what cost (62).

As social conditions may cause poor health, there is a hope that social prescribing would reduce the use of healthcare services and be cost-saving. This hope is attractive for healthcare funding organizations. However, findings to date are not clear-cut, as some found a reduction in GP visits and medical prescriptions (72, 73) and others found insignificant changes (74, 75). Part of these mixed-results is attributed to the fact that this scheme can also raise users' awareness of personal health status that consequently may increase medical care (6), and thereby may not be cost-saving from funder's perspective. Indeed, some experts think the current proliferation of this scheme is disproportionate to the evidence (62).

### Low Engagement of Clients

Low engagement of patients may be a major barrier. As social prescribing is relatively new, it is not easy for healthcare professionals to explain the process to patients. Pescheny et al. (76) found that entrenchment in medical solutions was a key reason for non-uptake of referrals to social prescribing by some patients. Patients expect healthcare professionals to perform an investigation, refer to a diagnostic center, and or write a prescription for a drug, not make a referral to social prescribing. Also, social prescribing does not prescribe the solution, but provides an atmosphere for patients to work on their plan of action. Moreover, it may take time to produce a tangible benefit. Furthermore, in reality, it is difficult to meet the varied needs of various clients, mainly due to the limited capacity of the facilities (53). Thus, clients' motivation for participation and engagement may be limited.

## Discussion

This paper outlines the impelling forces in favor of social prescribing and its challenges. Although this is an effort to apply a common knowledge, the recent growth of this scheme has the potential to bring the need for non-medical care into the forefront. The key ingredients that underpin this scheme and the idea of offering non-medical care in conjunction with medical care are sensible, and their benefits are understandable. However, although these sound simple, ensuring delivery of appropriate services for a reasonable duration and from local communities is not that straightforward. The challenge is not only to create services but also to ensure the key partners work together effectively.

Although having link-workers is a great help, healthcare professionals must still assess their patients in terms of suitability. Otherwise, there may have inappropriate referrals, long waiting periods, reduced referral-uptakes, and suspicion regarding the benefits of this scheme (54). In their study, White et al. (77) reported such problems with referrals for patients with severe mental health issues. It must be made clear that the scheme is not aimed so much at getting a problem solved as at helping people solve problems themselves (76, 78).

Although evidence for this scheme on health outcomes and healthcare costs are limited (79) and the findings to date are not very encouraging, some specific programs have shown to lead to improved health and wellbeing (80, 81). For instance, relatively good evidence exists around the benefits of arts and creative activities (82), and referrals to commercial providers for weight loss (83). This suggests that, if clients can be identified and referred properly, the scheme is likely to produce positive outcomes.

If the potential of social prescribing is to be realized, effective collaboration between the healthcare and the VCSE sector is vital. While primary care may be conservative to social prescribing, having skilled link-workers embedded and physical proximity between primary care and VCSE facilities may promote collaboration, make the “prescription” easier, and establish the provision of providing feedback to the healthcare professionals on patients' engagement/improvement that appears to be currently missing. A strong evidence base, up-to-date knowledge of local needs and services on offer (65, 67), and regular communication can be catalytic (18). Having a skilled link-worker embedded within the primary care.

Although the growing interest among healthcare leaders in addressing patients' non-medical needs indicates that they recognize the social model alongside the medical model (23), some are still uncomfortable about implementing non-medical care as a part of overall healthcare. Although academic content regarding the social aspects of health and health inequalities in the medical and allied health curriculum can help this scheme take root, and the concept “social determinants of health” is now included in the undergraduate medial and allied health curriculum in many settings (84), it is unclear how well this is delivered. Other endeavors, such as holding academic/professional conferences and allocating specific sessions on social prescribing in health conferences, can help to mainstream the scheme.

However, its long-term sustainability is a concern, mainly because of *ad hoc* nature of funding and the over-reliance on the voluntary sector. This scheme needs to stimulate cultural change; otherwise, as it gains political parlance it runs a risk of becoming a buzz-word with little real substance (85). Its sustainability also depends on the design of its service modality. Indeed, it might be practical to provide services only to a manageable cohort of patients rather than to everybody. The clients with complex needs may not be suitable under current arrangements. A balance is needed; otherwise, this scheme may fail if the target group is too broad (19) and unable to demonstrate tangible benefits if the target group is too narrow.

## Conclusion

The social prescribing narrative is compelling. There are several theoretical and practical factors in favor of this scheme. Indeed, social prescribing is a logical extension of the biopsychosocial model of healthcare. Therefore, the momentum for social prescribing is likely to be sustained, even with the lack of evidence to support its growth. From the primary healthcare perspective, this scheme presents an approach for expanding the avenue of social care for the patients. However, it will only see success when healthcare professionals fully accept it as a useful mechanism for improving the overall health and wellbeing of their patients.

## Data Availability Statement

The original contributions presented in the study are included in the article/supplementary material, further inquiries can be directed to the corresponding author/s.

## Author Contributions

MI conceived this study and wrote the paper.

## Conflict of Interest

The author declares that the research was conducted in the absence of any commercial or financial relationships that could be construed as a potential conflict of interest.
